# Circulating miRNAs in HER2-Positive and Triple Negative Breast Cancers: Potential Biomarkers and Therapeutic Targets

**DOI:** 10.3390/ijms21186750

**Published:** 2020-09-15

**Authors:** Ishita Gupta, Balsam Rizeq, Semir Vranic, Ala-Eddin Al Moustafa, Halema Al Farsi

**Affiliations:** 1 College of Medicine, QU Health, Qatar University, Doha P.O. Box 2713, Qatar; ishugupta28@gmail.com (I.G.); balsamr@gmail.com (B.R.); svranic@qu.edu.qa (S.V.); 2 Biomedical Research Centre, Qatar University, Doha P.O. Box 2713, Qatar

**Keywords:** circulating miRNAs, breast cancer, HER2-positive, triple negative breast cancer, receptor negative breast cancer, biomarkers

## Abstract

Breast cancer is one of the most prevalent diseases among women worldwide and is highly associated with cancer-related mortality. Of the four major molecular subtypes, HER2-positive and triple-negative breast cancer (TNBC) comprise more than 30% of all breast cancers. While the HER2-positive subtype lacks estrogen and progesterone receptors and overexpresses HER2, the TNBC subtype lacks estrogen, progesterone and HER2 receptors. Although advances in molecular biology and genetics have substantially ameliorated breast cancer disease management, targeted therapies for the treatment of estrogen-receptor negative breast cancer patients are still restricted, particularly for TNBC. On the other hand, it has been demonstrated that microRNAs, miRNAs or small non-coding RNAs that regulate gene expression are involved in diverse biological processes, including carcinogenesis. Moreover, circulating miRNAs in serum/plasma are among the most promising diagnostic/therapeutic tools as they are stable and relatively easy to quantify. Various circulating miRNAs have been identified in several human cancers including specific breast cancer subtypes. This review aims to discuss the role of circulating miRNAs as potential diagnostic and prognostic biomarkers as well as therapeutic targets for estrogen-receptor negative breast cancers, HER2+ and triple negative.

## 1. Introduction

Breast cancer is the second most common cancer worldwide, and it affects more than two million women accounting for around half a million deaths annually [1]. Breast cancer is a heterogeneous disease with several pathological features that are associated with disease prognosis and therapeutic options [2]. Microarray profiling classifies breast cancer into four molecular subtypes (luminal (A and B), HER2-positive and triple-negative breast cancer (TNBC)) with distinct gene expression patterns and clinical outcomes [3,4,5]; HER2-positive and TNBC subtypes are comparatively aggressive [6,7] and lack hormone receptors and hence are known as receptor negative breast cancers [8].

In comparison to other subtypes, HER2-positive breast cancer is characterized by the amplification/overexpression of HER2 protein and accounts for 15–20% of breast cancer cases [9,10]. The oncogene, *HER2 (ERBB2)* encodes a tyrosine kinase receptor which stimulates oncogenic pathways associated with increased cellular proliferation, invasion as well as angiogenesis. HER2-positive subtype is prone to lymph node metastasis and correlates with poor prognosis, short survival, and high rates of recurrence [6,10,11].

On the other hand, TNBC, lacks the expression of the three receptors (ER, PR and HER-2/neu); TNBC accounts for around 12–17% of all breast cancer cases [7]. Comparatively, TNBC commonly affects younger patients, and is characterized by advanced stage, higher Ki-67 proliferative index and advanced histologic grades [12,13,14,15]. In TNBCs, metastasis frequently occurs to visceral organs and bone [16,17,18]; in addition, cerebral metastasis is the most common [19,20,21].

Treatment for HER2-positive breast cancer involves targeted therapy using monoclonal antibodies, such as trastuzumab (Herceptin); which target the HER2 receptor and inhibit its associated pathways regulating proliferation, survival, migration and cell invasion [22]. Other available treatments include lapatinib [23], pertuzumab, ado-trastuzumab emtansine (T-DM1), trastuzumab deruxtecan (DS-8201), neratinib, and tucatinib [24,25].

Since TNBC cells are deficient in ER, PR and HER2 receptors, treatment options are presently limited; hence, cytotoxic chemotherapy is the standard approach for TNBCs thus far [26,27]. As such, new therapeutic options for this group of breast cancers are highly needed. In this regard, recent studies have shown great promise by targeting *PD-L1* receptors using immune checkpoint inhibitors. This is particularly important as PD-L1 expression is present in advanced TNBCs, thus providing the opportunity for treatment with immunomodulatory drugs or anti-PD-1/PD-L1 therapies [28,29,30,31].

Nevertheless, although estrogen-receptor negative breast cancer initially responds well to therapy, it generally becomes resistant with higher rate of recurrence compared to other subtypes of breast cancers, resulting in tumor progression and death in around 90% of patients with advanced/metastatic disease [32,33].

On the other hand, several recent studies have shown that microRNAs (miRNAs) can provide a potential diagnostic and therapeutic approach for human breast cancers including estrogen-receptor negative breast cancers [34,35,36]. Moreover, circulating miRNAs can be used as a non-invasive tool for early diagnosis and provide a new avenue for estrogen-receptor negative breast cancer management [37]. In this review we will summarize the recent advances made in the identification and role of estrogen-receptor negative specific circulating miRNAs.

## 2. Circulating miRNAs (ci-miRNAs)

**Figure 1:**
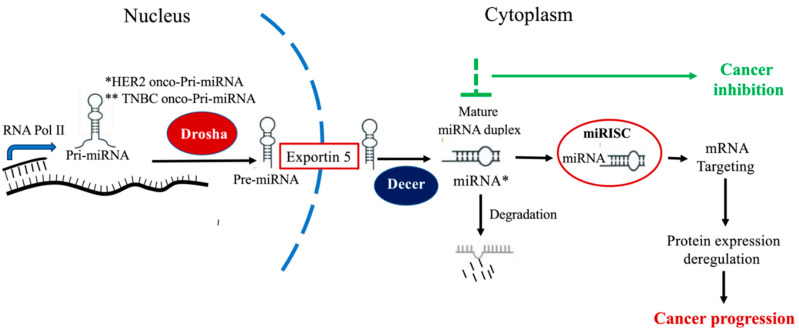
Schematic outline of miRNA biogenesis in “*HER2 and **TNBC” cells. Synthesis of primary miRNA (pri-miRNA), by RNA pol II precedes sequential nuclear processing by the microprocessor complex containing Drosha. Then, export of miRNA precursor (pre-miRNA) to the cytoplasm is carried out via Exportin 5 followed by removal of the loop region in the pre-miRNA by an endonuclease complex containing Dicer to generate a mature miRNA/miRNA* duplex. The mature miRNA strand is then selectively loaded onto miRISC complexes where miRNA-directed targeting of mRNAs will take place leading to specific proteins expression deregulation which can play a role in stimulating cancer progression, whereas the miRNA* strand is preferentially degraded. Thus, blocking these miRNAs may lead to inhibition of cancer progression.

MiRNAs belong to the class of small non-coding RNAs, measuring around 25nt in length. miRNAs have structural, regulatory as well as catalytic functions at the post-transcriptional level [38,39,40]. While, miRNAs can degrade or inhibit mRNA translation by targeting 3′ untranslated region (UTR), they are also known to target the 5′ UTR, gene promoters and coding sequences [41,42]. While, miRNA synthesis process is tightly controlled; dysregulation of miRNA expression alters several cellular processes including transcription, signal transduction, proliferation, growth, differentiation as well as apoptosis [43,44,45], thus, indicating their role in the onset and progression of cancer. In addition, miRNAs mediating metastasis have been identified and act as metastasis activators or suppressors [46,47]. These miRNAs play a vital role in metastatic cascade, including migration, invasion, adhesion, epithelial-mesenchymal transition (EMT), extracellular matrix modification and proliferation at the distant site. During carcinogenesis, miRNAs can act as both, oncomiRNAs (Figure 1) as well as tumor suppressors [48,49,50,51].

Since, miRNAs play a vital role in cancer initiation and progression, there is a wide spectrum of latent applications of miRNA measurements in oncology. Discovery of miRNAs in biofluids has paved the way for their role as biomarkers in the diagnosis of various diseases including neurodegenerative [52], cardiovascular diseases [53], diabetes [54] and cancer [55]. Moreover, in comparison to normal tissues, tumor samples from the same patient differentially express miRNAs [56,57]; unique miRNA expression patterns are present in several cancers such as prostate [58], colon [59], gastric [60], ovary [61] and breast [62]. Stability of miRNAs facilitates their detection in circulation, thus, circulating miRNAs can serve as potential biomarkers that can be measured repeatedly and non-invasively in several types of cancer [63].

Cell-free circulating miRNAs (ci-miRNAs) were initially identified by Chim et al. (2018), as they noted that miRNAs are differentially expressed in the placenta and plasma of pregnant women [64]. Cell-free ci-miRNAs facilitate cellular signaling and are presently considered as plausible biomarkers for several diseases [65]. Ci-miRNAs are of two kinds, vesicle-associated and non–vesicle-associated, which suggest the distinct mean of their secretion. While, the majority of ci-miRNAs are present in a non–vesicle-associated form (ribonucleoprotein complex) [66,67,68], membrane-bound vesicles (exosomes and microvesicles) are isolated and purified from plasma/serum [69,70,71].

Depending on their origin, ci-miRNAs play a role in various cellular processes and target gene expression in recipient cells, thus, favoring cellular differentiation, proliferation as well as apoptosis [72]. Several investigations attempted to elucidate the underlying mechanism of ci-miRNA release, packaging and uptake, some suggesting that the process may involve selective packaging and release mechanisms [73]. In general, methods of miRNA release into circulation include exosomes/microvesicles, L-exosome, lipoproteins, and RNA-protein complex in addition to release by damaged cells due to necrosis or inflammation [63,67,74,75]. In general, miRNAs are selectively packaged into exosomes and become a critical part of the disease microenvironment; which is one of the underlying mechanisms of ci-miRNAs tissue/disease specificity. For instance, post-injury, ci-miRNAs- 21, -122, -192, -200c, -208 and -423 are generally released from the heart, liver and kidney [76,77,78,79]. While, other ci-miRNAs are found to be involved in the onset and development of tumor progression as well as in the invasion and metastasis of cancer [80]. In this regard, release of immunosuppressive miRNAs allows cancer cells damage by T and B cells; on the other hand, angiogenic miRNAs allow cancer cells to recruit capillary blood vessels, thus promoting inflammation and angiogenesis. Moreover, it has been pointed out that dysregulation of ci-miRNA expression significantly enhances growth and spread of cancer cells [81,82,83]. MiRNAs released from tumor cells and immunocytes act synergistically ensuing poor clinical outcomes [84,85,86]. More importantly, ci-miRNAs can be associated with specific tumor progression stages as they are differentially expressed during different stages of cancer, thus, playing a vital role in carcinogenesis and its progression [87]. Based on these studies it is clear that ci-miRNAs could be used as promising biomarkers and therapeutic targets for different types of cancers including estrogen-receptor negative breast cancers (Figure 1).

## 3. Circulating miRNAs in HER2-Positive Breast Cancer

MiRNAs play a vital role in controlling gene expression and therefore they are implicated in breast carcinogenesis [88]. MiRNAs are stable in blood, plasma, and serum and can be detected in circulation [63]. Recently, ci-miRNAs are being studied in breast cancer patients as promising diagnostic, predictive and prognostic biomarkers for the development of therapeutic strategies [89]. Identification of ci-miRNAs can serve as non-invasive and cost-effective biomarkers to help identify high-risk patients [90].

An earlier investigation by Fan et al. (2018) applied branched rolling circle amplification (BRCA) assay to analyze sera levels of ci-miRNAs as a means to detect early breast cancer. They were able to identify ci-miRNAs-16, -21, -155, and -195 in early breast cancer patients in comparison with healthy controls [91]. More recently, a study of breast cancer patients sera showed post-surgery loss of ci-miRNAs-130b-5p, -151a-5p, -206, and -222-3p compared to breast cancer group, where their expression were significantly elevated, thus implicating the important diagnostic and prognostic role for these five ci-miRNAs [87]. The study also reported that patients expressing three or more of these ci-miRNAs have shorter disease-free survival compared to those with 0–2 highly expressed ones [87]. Interestingly, the authors identified miR-222-3p as a candidate biomarker for the early detection and prognosis prediction of breast cancer [87]. Furthermore, qPCR analysis of breast cancer patient plasma samples reveal considerably elevated levels of ci- miRNA-21 and ci-miRNA-146a in breast cancer patients in comparison with healthy controls indicating their diagnostic potential for breast cancer [92]. Additionally, ci-miRNA-21 is significantly associated with lymph node involvement, metastasis, high tumor grade and advanced clinical stage [93,94,95], thus, confirming its role as a diagnostic and prognostic marker [93].

Several recent studies have shown that ci-miRNAs are present in breastmilk and are associated with mammary gland infections (mastitis) [96] as well as cancers [97,98,99] including breast cancer [73,97,100]. Interestingly, numerous investigations that identified ci-miRNAs in breastmilk concluded that they are immunity-modulating microRNAs [101,102,103,104,105]; few of them found that ci-miRNAs are differentially expressed in-vitro [73] and in-vivo [97] during the onset of breast cancer [106]. Ci-miRNAs-509-5p, -515-3p, and -335 were the most profuse ci-miRNAs in the majority of body fluid samples including milk, however, ci-miRNA-193b was unique to breastmilk [107]. Moreover, Zhou et al., identified 10 ci-miRNAs (-148A-3P, -30B-5P, let-7f-5p, -146B-5P, -29A-3P, let-7a-5p, -141-3P, -182-5P, -200A-3P and -378-3P) in breastmilk [105]. Similarly, Munch et al., also found ci-miRNAs-148a, -200c, -146b-5p, -30d, 21, -103, let-7b, let-7g, let-7a and let-7f in breastmilk [108]. Based on these studies and others, tumor suppressor let-7 family of miRNAs as the most common ci-miRNAs in milk from healthy patients; however, their expression was lost in patients with breast cancer as well as milk stasis, thus indicating a possible role of milk stasis in the onset of breast cancer [100,109,110,111]. While, ci-miRNA-181a was detected commonly in serum and breastmilk, some of the ci-miRNAs are found to be differentially expressed in different biological fluids of breast cancer patients in comparison with healthy controls [97]. Real-time PCR analysis identified the presence of ci-miRNAs-140, -21 and let-7a in breastmilk in higher abundance compared with blood [100]. However, data correlating the presence of ci-miRNAs in breastmilk and breast cancer is scarce and requires further investigations.

Ci-miRNAs are associated with HER2-positive breast cancers. Comprehensive de novo sequencing, of breast cancer patients’ sera show significant association of ci-miRNAs-375 and -122 with clinical outcomes; elevated levels of ci-miRNA-122 is specifically predictive of metastatic recurrence in early stage (II-III) breast cancer patients [112]. Moreover, the study also analyzed differentially expressed ci-miRNAs in HER2-positive and HER2-negative breast cancer patients; elevated ci-miRNA-375 and reduced ci-miRNA-122 levels correlate with positive HER2 status, response to neoadjuvant chemotherapy and absence of relapse [112]. A recent report exhibited a differential expression of exosomal miR-101 and miR-373 in patients with breast cancer and benign breast tumors; exosomal miR-101 serum levels are deregulated in HER2-positive breast cancer in comparison to its levels in healthy women [113]. A recent study also analyzed ci-miRNAs in serum of breast cancer patients to identify subtype-specific molecular profiles of cell-free miRNAs for early detection of breast cancer in serum; the NanoString platform identified the 42 significant differentially expressed ci-mi-RNAs in each molecular subtype [114]. Of these 42, only four ci-miRNAs (ci-miRNAs- 548ar-5p, 584-3p, 615-3p and 1283) are expressed in the HER2-positive subtype [114].

Moreover, ci-miRNAs can potentially identify patients with differential response to HER2-targeted therapy [115]. A recent study conducted by Di Cosimo et al. (2020) used high-throughput analysis of 752 miRNA assays from HER2-positive breast cancer patients to identify differentially expressed ci-miRNAs before and after two weeks of trastuzumab administration [115]. The study reported enhanced levels of ci-miRNA-148a-3p and ci-miRNA-374a-5p, which are significantly associated with pathological complete response [115]. The same group analyzed ci-miRNAs as noninvasive biomarkers to predict efficiency of single/dual HER2-targeted therapy in the Neo ALTTO (Neoadjuvant Lapatinib and/or Trastuzumab Treatment Optimization) study. They found that levels of ci-miRNA-140-5p after two weeks of treatment correlate with prognosis in trastuzumab-responsive patients as well as event-free survival [116]. Another report found significant association between a panel of 3 ci-miRNAs (ci-miRNA-21, ci-miRNA-210, and ci-miRNA-373) and poor clinical outcome in HER2-positive non metastatic breast cancer patients treated with neo-adjuvant therapy, or adjuvant therapy ± trastuzumab [117]. Furthermore, ci-miRNA-210 was thought to be a predictive factor of response to trastuzumab, tumor burden, and lymph node metastases [118]. On the other hand, a 2-ci-miRNA (miR-4734 and miR-150-5p) based signature is identified as a dependable prognostic biomarker for HER2 positive breast cancer patients [119]. Li et al. (2018) employed miRNA microarray and identified 13 differentially expressed ci-miRNAs in the serum of HER2-positive metastatic breast cancer patients with distinct response to trastuzumab; of these 13, four ci-miRNAs (ci-miRNAs-940, -451a, -16-5p and -17-3p) can predict trastuzumab therapeutic benefit in HER2-positive metastatic breast cancer patients [120].

Interestingly, Stevic et al. (2018) aimed to identify unique ci-miRNAs from plasma to distinguish HER2-positive breast cancer from TNBC compared with healthy women [121]. In comparison to the control, the levels of ci-miRNA-27a and ci-miRNA-365 are only significantly enhanced in HER2-positive patients, but not in TNBC patients [121]. On the other hand, ci-miR-27b is enhanced in both subtypes; however, comparatively higher exosomal levels have been detected in HER2-positive patients; HER2 triggers miR-27b expression via the AKT/NF-κB signaling cascade [121,122]. Furthermore, high exosomal miR-27b is found to predict pathological complete response in HER2-positive patients [121]. On the other hand, levels of exosomal miR-422a are decreased in HER2-positive breast cancer patients when compared to TNBC patients [121]. Based on clinicopathological characteristics, there is a significant correlation between ci-miRNAs-16, -328 and -660 and lymph node status in HER2-positive subtype but not in TNBC patients; additionally, ci-miRNA-660 can be used to predict the pathological response to neoadjuvant therapy in HER2-positive patients [121]. Similarly, 6 ci-miRNAs (miR-185, miR-376a, miR-382, miR-410, miR-433, and miR-628) are associated with higher tumor size in HER2-positive breast cancer patients [121]. Microarray profiling and validation by qPCR in plasma from breast cancer patients identified a significant upregulation of ci-miRNA-107 in ER-negative patients compared with ER-positive patients [123]. Furthermore, plasma levels of miRNA-130a and miRNA-146a are significantly upregulated in HER2-positive breast cancer patients [123].

Table 1 summarizes key ci-miRNAs with their expression levels and biological functions in HER2-positive breast cancer.

## 4. Circulating miRNAs in TNBC

Today, with important advances made in genomic profiling more comprehensive screening of genetic signatures and their downstream targets is possible. In this context, a study by Souza et al. (2019) using NanoString platform was able to identify and quantify 42 differentially expressed ci-miRNAs in the serum of breast cancer patients [114]. Among these ci-miRNAs, the expression of miRNA-25-3p was elevated in TNBC patient serum in comparison with matched healthy controls [114]. Moreover, it has been revealed that ci-miRNAs-18b, -103, -107 and -652 significantly correlate with tumor recurrence and reduced survival in TNBC patients [126]. Furthermore, elevated levels of ci-miRNAs-21-5p, -375, -205-5p, -194-5p and reduced expression of ci-miRNas-382-5p, -376c-3p, -411-5p are also associated with recurrent breast cancer cases [127]. Moreover, upregulated expression of ci-miRNAs-21, -210 and -221 are found to be significantly involved in the onset and progression of TNBC [128].

A panel of four ci-miRNAs were identified in a recent miRNA profiling of TNBC patients that could be used as both diagnostic and prognostic biomarkers; three ci-miRNAs (miRNA-376c, miRNA-155 and miRNA-17a) are reported as early stage biomarkers, while, ci-miRNA-10b is found as late stage biomarker of TNBC [129]. Similarly, in the study by Stevic et al. (2018) significantly higher levels of exosomal miRNA-376c, miRNA-382 and miRNA-433 are observed in TNBC patients, but not in HER2-positive breast cancer patients [121]. Moreover, ci-miRNA-374 significantly correlates with a higher tumor size in TNBC patients [121]. Another investigation showed that a four serum miRNA signature (miRNA-16, miRNA-25, miRNA-222 and miRNA-324-3p) correlates with enhanced risk of breast cancer thus providing a non-invasive predictive biomarker [130]. The study also demonstrated that a combination of miRNAs-484 and -191 can be used as endogenous control for serum miRNA detection [130]. MiRNA profiling of TNBC patients from Metabric database revealed high expression of ci-miRNA-105 and ci-miRNA-93-3p in TNBC patients which is significantly associated with poor survival [131]. More specifically, it has been reported that miR-105/93-3p stimulates Wnt/β-catenin signaling by reducing SFRP1 levels, which is associated with chemoresistance and metastasis in TNBC cells [131]. Notably, combination of ci-miRNA-105/93-3p levels indicate the presence of a potential diagnostic biomarker for both early- and late-stage TNBC [131].

A comparative study between ci-miRNAs in TNBC and non-TNBC patients using miRCURY LNA array platform and real-time PCR showed that loss of ci-miRNA-199a-5p correlates with disease stage, thus, suggesting its role as a plausible TNBC-specific diagnostic marker [90]. An investigation by Chen et al. (2016), further explored the underlying mechanism of ci-miRNA-199a-5p in TNBC revealing its role in EMT and blocking breast cancer cell stemness by reducing CD24-/CD44+ expression and ALDH activity [132]. TaqMan-based miRNA profiling in tumor, adjacent non-tumor, corresponding plasma from breast cancer patients, and plasma from matched healthy controls identified upregulation of miRNAs-16, -21 and -451 and downregulation of miRNA-145 in plasma of breast cancer patients [62]. Additionally, this study demonstrated a combination of miRNA-145 and miRNA-451 as a candidate biomarker for breast cancer in comparison with healthy controls and other types of cancers [62]. On the other hand, analysis by qPCR in TNBC patients revealed that the expression of ci-miRNA-34a/34b/34c is significantly reduced in TNBC patients compared to controls; while, low miRNA-34a/c expression correlate with tumor progression and worse prognosis and miRNA-34b is significantly associated with lymph node positivity [133]. More recently, a study by Ozawa et al. (2020), analyzed the expression of ci-miRNAs in breast cancer subtypes revealing that elevated ci-miRNA-142-5p expression distinguish luminal A subtype from TNBC, however, the accuracy was increased in combination with ci-miRNA-320a [134]. Moreover, ci-miRNAs expression was analyzed in a cohort of TNBC patients in comparison with estrogen and progesterone positive patient group (ER/PR); they concluded that ci-miRNA-200c expression is reduced in TNBC patients compared with the ER/PR positive group [135].

Zhu et al. (2018) analyzed the association of ci-angiogenic miRNAs and the incidence of cardiotoxicity in TNBC patients treated with neoadjuvant chemotherapy [136]. The study showed that in comparison with non-cardiotoxic patients, levels of ci-miRNAs- let-7f, -19a, -20a, -126, and -210 are reduced [136]. Further validation by RT-qPCR suggested use of let-7f and miRNA-126 as candidate biomarkers for cardiotoxicity risk in TNBC patients undergoing EC-D neoadjuvant chemotherapy [136]. Similarly, a study in Saudi female breast cancer patients, showed upregulation of ci-miRNA-let-7f in luminal breast cancer, while, ci-miR-195 is elevated in TNBC patients [137]. Eichelser et al. (2014) explored the expression of ci-miRNAs in blood sera of breast cancer patients using quantitative TaqMan MicroRNA assays; according to their findings, cell-free miRNAs- 101 and -373 can be used as breast cancer-specific markers, additionally, sera levels of exosomal (not cell-free) miR-373 are significantly higher in the TNBC subtype and in ER-/PR− breast cancer patients, thus, suggesting a correlation between miR-373 and negative receptor status [113]. Functional assays demonstrated that miRNA-373 reduces ER expression and induces cell growth and proliferation by camptothecin (a topoisomerase poison) [113].

Table 2 summarizes the key ci-miRNAs with their expression levels and biological functions in TNBC.

## 5. The Role of ci-miRNAs in Cellular Signaling Pathways

Several studies have shown that ci-miRNAs play a crucial role in dysregulating gene expression via several major pathways involved in inhibiting apoptosis and inducing breast cancer invasion and metastasis [142,143]. Pathway analysis of differentially expressed ci-miRNAs have indicated plausible cellular signaling pathways playing a vital role in breast cancer including TGF-β, p53, MAPK/ERK and Wnt signaling pathways [143,144,145].

In metastatic breast cancer, upregulated ci-miRNA-181a expression levels can be controlled by TGFβ-SMAD signaling pathway, which further enhance the expression of the transcription factor, SNAIL-1 and inhibit Bim expression, thus, promoting breast tumor aggressiveness and metastasis via EMT [146]. Similar to ci-miRNA-181a, ci-miR-18a inhibit breast cancer induced lung metastasis by targeting SREBP1, which forms a SNAIL/HDAC1/2 complex to modulate EMT [147]. Moreover, ci-miRNA-200c expression is lost during EMT, therefore it is considered one of the hallmarks of breast cancer metastasis [148,149]. Ci-miRNA-200c family members control the expression of BMI1 in breast cancer cells and inhibit ZEB1/2 expression, thus reducing EMT [150,151]. Furthermore, miRNA-200c regulates, TGF-β [144]; which is known to stimulate bone and lung metastases [152,153]. Furthermore, studies by Iorio et al., and Yan et al. showed that TGF-β1 and the receptor TGFβR2 are target genes of miRNA-21, indicating a role of TGF-β in mediating miRNA-21-induced breast cancer [154,155]. Ci-miRNA-21 has several targets involved in breast tumorigenesis including *PDCD4*, *TPM1* [156,157] and *PTEN* [158]. In breast cancer cells, overexpressing ci-miRNA-191, was associated with upregulated levels of TGF-β pathway genes (TGFβ2, SMAD3, BMP4, JUN, FOS, PTGS2, CTGF, and VEGFA), thus promoting breast cancer growth and metastasis [159]. On the other hand, ci-miRNA-34a, is a key member of the p53 group; therefore, loss of both ci-miRNA-34a and p53 enhances tumor growth and promote breast cancer progression [145]. Several studies also showed that inducing the expression of miRNA-34a in breast cancer cells promote apoptosis and inhibit tumor suppression by increasing Bcl-2 and SIRT1 levels [145,160]. While, ci-miRNA-10b expression is enhanced via the transcription factor, twist and targets Krüppel-like factor 4 genes, which further inhibits the expression of *HOXD10* and enhances the expression of pro-metastatic *RHOC* gene leading to tumor invasion and metastasis [161,162]. Moreover, Let-7 targets GTPase HRas and the high mobility group A2 (HGMA2), thus, regulating breast tumor-initiating cells [110].

Ci-miRNAs are also involved in regulating Wnt/β-catenin and Notch signaling pathways, thus playing a vital role in controlling EMT and tumor metastasis/progression [142]. In this regard, elevated levels of ci-miRNA-374a inhibit the expression of epithelial markers (E-cadherin), and promote the expression of mesenchymal markers (N-cadherin and vimentin), thus enhancing EMT [163]. Moreover, miRNA-374a activates the Wnt/β-catenin pathway by promoting nuclear translocation of β-catenin and targets negative regulators of the signaling pathway including PTEN and WNT5A [163]. Also, loss of ci-miRNA-148a targets WNT1, a ligand of the Wnt/β-catenin pathway; thus, promoting breast cancer migration, invasion and metastasis [164]. On the other hand, loss of ci-miRNA-148a was found to promote breast cancer cell proliferation, colony formation, and tumor angiogenesis by targeting IGF-IR and IRS1 and upregulating their downstream AKT and MAPK/ERK signaling pathways [165]. On the other hand, loss of ci-miRNA-101 enhance the expression of Notch signaling pathways components including jagged1, Hes1, and Hey1, which results in apoptosis inhibition [166]. Additionally, loss of ci-miRNA-101 levels in breast cancer, inhibit apoptosis by upregulating SOX2 levels, thus, promoting breast cancer growth, proliferation and migration [167]. Downregulated ci-miRNA-424 expression in breast cancer cells enable the proliferation and regulation of cell cycle by allowing continuous cell accumulation in the G2–M cell phase [168]. Furthermore, ci-miRNA-424 act as a tumor suppressor by binding to cyclin-dependent kinase *CDK1* while reduced ci-miRNA-4242 levels enhance the expression of YAP and p-ERK1/2 of the Hippo and ERK pathways, respectively [168].

## 6. Ci-miRNAs as Potential Biomarkers and Therapeutic Targets-Advantages and Limitations

As mentioned earlier, ci-miRNAs released from primary tumors and surrounding cells are present in circulating body fluids. Nevertheless, although ci-miRNAs can be detected in virtually all body fluids (blood, serum, plasma, urine, breastmilk and saliva) [107], the amount of ci-miRNAs present in each fluid type varies, with the highest levels present in saliva, blood, breastmilk, and seminal fluid and the lowest in human urine [63,69,107,169,170]. Thus, ci-miRNAs are considered as a potential, non-invasive diagnostic and prognostic biomarkers for various diseases including cancer [170,171,172]. Furthermore, ci-miRNAs are easier to identify due to their locations upstream in signaling cascades.

Early detection and diagnosis in HER2-positive breast cancer and TNBC patients aids in providing a more favorable outcome in diseases management [173]. Ci-miRNAs are associated with clinical and pathological features and can target different genes and influence several signaling pathways, thus, indicating their role as plausible candidate diagnostic, prognostic as well as predictive breast cancer biomarkers [174]. Due to certain basic features, ci-miRNAs are ideal candidates for non-invasive cancer biomarkers [63,75,175]. These studies clearly indicate the importance of ci-miRNAs as non-invasive tools in the early diagnosis of breast cancer and the classification of its subtypes, as well as their role as potential therapeutic targets especially for oncomiRNAs (Figure 1).

Nevertheless, the idea of developing an accurate clinical panel of ci-miRNAs as cancer biomarkers poses several challenges; from the first step of sample collection through to processing and data analysis. Since ci-miRNAs have low concentrations, it is often difficult to measure their expression through miRNA profiling/qRT-PCR/microarrays [176]. Another foremost limitation in miRNA identification and analysis in body fluids is the need for an appropriate housekeeping gene for internal normalization [177]. Certain housekeeping genes, often used as internal normalization during miRNA analysis in various cells and tissues, are not viable in serum analysis since these controls are easily degraded and not detected in serum [177]. Indeed, ci-miRNA expression values varies between sera and plasma samples taken from the same individual, as total RNA concentration is generally higher in the serum than in the plasma, possibly due to the RNA released from blood cells and platelets [178]; This indicates that preliminary sample collection is a vital issue and specific care should be taken to exclude hemolyzed samples. Moreover, to overcome conflicting findings reported in studies, patient selection with appropriate clinical diagnosis is important, as they influence results derived from clinical data [170,179]. In addition to the importance of establishing proper norms for sample selection, the isolation technique is another area of concern. Identifying ci-miRNAs specific to breast cancer would require augmenting ci-miRNAs during isolation, which would further aid to differentiate tumor origin biomarkers. Moreover, their underlying mechanisms in oncogenesis/metastasis (Figure 1), expression in specific types of cancers, different tumor stages as well as in response to treatment are still unclear and warrant further investigation [176]. With respect to targeted therapy, few limitations are present, one of them being, restricted tissue specificity and permeability and hence, nanoparticles carrying miRNAs are now being designed for improved specificity and reduced immunotoxicity [180]. Additionally, in-vivo studies demonstrated that packaged artificial and modified miRNAs in exosomes enhance stability of miRNAs [181]. Nonetheless, preclinical studies involving animal models should be considered to validate the efficiency of blocking ci-oncomiRNAs in cancer therapy (Figure 1).

Although several studies have identified and reported various ci-miRNAs in breast cancer, there is lack of significant inferences with respect to identification of a single ci-miRNA panel to aid in breast cancer diagnosis. This discrepancy can be attributed to breast cancer heterogeneity and its complex biological behavior and clinical presentation.

## 7. Conclusions

Estrogen-receptor negative breast cancers (HER2+ and TNBC) have higher mortality rate, which necessitates the discovery of new specific biomarkers as both predictive and potential therapeutic targets. MiRNA analysis, especially its circulating form, as biomarkers for breast cancer is a rapidly evolving field that carries a lot of promise for early non-invasive diagnosis that can be easily integrated in routine blood tests and patient follow ups. Recent investigations have also shown that ci-miRNAs are detectable and differentially expressed in the plasma, thus indicating their role as favorable diagnostic biomarkers for breast cancer. Nevertheless, there are still a lot of challenges in introducing this novel promising breast cancer diagnosis tool; therefore, further studies are essential to determine their clinical utility, not to mention their possibly important role as oncogenes, in the development of novel human breast cancer therapeutic avenues, which can pave the way for more specific targeted approaches.

## Figures and Tables

**Table 1:** List of ci-miRNAs and their roles in HER2-positive breast cancer.

Biological Functions	Circulating-miRNAs		References
	Stimulate	Inhibit	
Drug Resistance and Tumor Relapse	miRNA-21/210/373, miRNA—16-5p/17-3p/451a/940, miRNA-26a-5p/106b-5p/150-5p/155-5p/205-5p/361-5p/365a-3p	miRNA-148a-3p/374a-5p, miRNA-140-5p, miRNA-375/122, miRNA-27b/660, miRNA-424-3p/4734	[112,115,116,117,119,120,121]
Cell Migration and Proliferation	miRNA-27a/365/382, miRNA-130a	miRNA-628/598/422a, miRNA-146a, miRNA-107	[121,123,124]
Cell Apoptosis	miRNA-628/598/422a	miRNA-382	[121]
Angiogenesis	miR-27a		[121,125]
High Tumor Size	miRNA-185/376a/382/410/433/628		[121]
Tumor Metastasis and Progression	miRNA-27a/301/365/628, miRNA-107/130a/146a	miRNA-422a/598, miRNA-146a, miRNA-107	[121,123,124]
EMT	miRNA-382		[121]

**Table 2:** List of ci-miRNAs and their roles in triple-negative breast cancer (TNBC).

Biological Functions	Circulating-miRNAs		References
	Stimulate	Inhibit	
Drug Resistance and Tumor Relapse	miRNA-105/93-3p, miRNA-18b/103/107/652, miRNA-202, miRNA-21/210/221/222, miRNA-130a-3p/451a, miRNA-21-5p/375		[126,127,128,131,138,139]
Cell Migration and Proliferation	miRNA-101, miRNA-25-3p, miRNA-21-5p, miRNA-17a, miRNA-142-5p	miRNA-199a-5p, miR-891a-5p	[90,113,114,121,127,129,140]
Cell Apoptosis	miRNA-142-5p	miRNA-373, miRNA-let-7f/126	[113,136,140]
Angiogenesis	miRNA-let-7f/126		[136]
High Tumor Size	miRNA-374		[121]
Tumor Metastasis and Progression	miRNA-34a/c, miRNA-155, miRNA-200c, miRNA-21/210/221, miRNA-17a/376c	miRNA-628	[121,128,129,133,135,141]
EMT	miRNA-199a-5p, miRNA-375, miRNA-17a		[90,129,132]

## Abbreviations

BMI1	B cell-specific Moloney murine leukemia virus integration site 1
ci-miRNA	Circulating-Micro-Ribonucleic Acid
EMT	Epithelial-Mesenchymal Transition
ER	Estrogen Receptor
HER2	Human Epidermal Growth Factor Receptor 2
HOXD10	Homeobox 10
miRNA	Micro-Ribonucleic Acid
Neo ALTTO	Neoadjuvant Lapatinib and/or Trastuzumab Treatment Optimisation
PDCD4	Programmed cell death 4
PD-L1	Programmed Death-Ligand 1
PR	Progesterone Receptor
PTEN	Phosphatase and tensin homolog
RHOC	Ras homolog family member C
SIRT1	Sirtuin 1
TGF-β	Transforming growth factor-β
TNBC	Triple Negative Breast Cancer
TPM1	Tropomyosin 1α
UTR	Untranslated Region
YAP	Yes associated protein
ZEB	Zinc-finger E-box binding homeobox
